# Active endocannabinoids are secreted on the surface of microglial microvesicles

**DOI:** 10.1186/2193-1801-4-S1-L29

**Published:** 2015-06-12

**Authors:** Martina Gabrielli, Natalia Battista, Loredana Riganti, Ilaria Prada, Flavia Antonucci, Laura Cantone, Marta Lombardi, Michela Matteoli, Mauro Maccarrone, Claudia Verderio

**Affiliations:** Department of Medical Biotechnology and Translational Medicine, University of Milano, Milano, Italy; Faculty of Bioscience and Technology for Food, Agriculture and Environment, University of Teramo, Teramo, Italy; CNR-IN Neuroscience Institute, Department of Medicine, Milan, Italy; European Center for Brain Research/IRCCS Santa Lucia Foundation, Rome, Italy

**Keywords:** Endocannabinoids, extracellular vesicles, microglia-neuron signaling

Endocannabinoids (eCBs) are bioactive lipids which primarily influence synaptic communication in the nervous system. They are synthesized by neurons but also by microglia, especially under inflammation. To exert their function, eCBs travel across the intercellular space. However, how eCBs move extracellularly remains obscure. Our recent evidence indicates that reactive microglia release extracellular vesicles (EVs), which may represent an ideal vehicle for the transport of hydrophobic eCBs. Hence, in this study we investigated whether microglial EVs carry eCBs and may influence neurotransmission. First we analyzed the eCB content of EVs and found a clear enrichment of anandamide (AEA) in EVs relative to parental microglia. This analysis revealed higher AEA levels in EVs shed from the plasma membrane (microvesicles), compared to those which originate from the endocytic compartment (exosomes). To bioassay the activity of vesicular AEA, we used patch clamp analysis of miniature inhibitory post-synaptic currents (mIPSC) on hippocampal neurons in vitro. Exposure of neurons to microvesicles (MVs) induced a significant decrease in mIPSC frequency, mimicking the action of CB1R agonists. The involvement of vesicular AEA in this phenomenon was inferred from the ability of the CB1R antagonist SR141716A to block the reduction of mIPSC frequency evoked by MVs. Western blot analysis showed that MVs induces an increase in ERK phosphorylation, which was completely inhibited by SR141716A. This indicate that CB1R activation by AEA-storing MVs translates into downstream signaling. Finally, consistent with a surface localization of AEA, MVs membranes maintained their capability to decrease mIPSC frequency. Overall, this study shows that microglial MVs carry AEA on their surface to stimulate CB1R on target GABAergic neurons thus playing a crucial role in the modulation of inhibitory transmission.
